# Association of hospital/ICU characteristics with HAIs: findings from the SPIN-UTI project

**DOI:** 10.1093/eurpub/ckac129.153

**Published:** 2022-10-25

**Authors:** M Barchitta, A Maugeri, G Favara, R Magnano San Lio, C La Mastra, MC La Rosa, E Campisi, I Mura, A Agodi, SPIN-UTI network

**Affiliations:** 1 Department GF Ingrassia, University of Catania, Catania, Italy; 2 GISIO, SItI, Rome, Italy; 3 University of Sassari, Sassari, Italy

## Abstract

**Background:**

Preventing the spread of healthcare-associated infections (HAIs) in Intensive Care Units (ICUs) constitutes a priority for Public Health. In a country with decentralized healthcare system, the comparison between and within regions might represent a useful approach to identify what hospital and ICU indicators are associated with HAIs.

**Methods:**

Using data from the SPIN-UTI (”Sorveglianza attiva Prospettica delle Infezioni Nosocomiali nelle Unità di Terapia Intensiva”) network, the present analyses aimed to identify the main hospital and ICU indicators associated with HAI incidence at national level, and to stratify the analyses between Italian regions.

**Results:**

No associations between hospital/ICU characteristics and HAIs were evident at national level. However, ICUs in Southern Italy showed the highest incidence density of HAIs if compared with those in Central and Northern Italy (p < 0.001). Stratified analyses found a positive association of incidence density of HAIs and total days in ICU in Northern Italy (β = 0.3; SE = 0.1; p = 0.002); a positive associations with ICU size (β = 1.8; SE = 0.7; p = 0.020), total days in hospital (β = 0.06; SE = 0.02; p = 0.037) and total days in ICU (β = 0.5; SE = 0.1; p = 0.006) in Center Italy; a positive association with hospital size in Southern Italy (β = 20.3; SE = 9.4; p = 0.033).

**Conclusions:**

Although our study confirms that HAIs still represent an important issue in Italian ICUs, there is some variation between regions from Northern, Central and Southern Italy. In general, we found that HAI incidence increased with increasing number of beds in hospital and in ICU, as well as with the the increasing number of patient-days. However, further research is necessary to better understand if additional hospital and ICU characteristics could motivate the observed regional differences.

**Key messages:**

• There is a large regional variation in the incidence of HAIs in Italian ICUs and hospitals.

• This difference that could be motivated by specific hospital and ICU characteristics.

